# BioMAX – the first macromolecular crystallography beamline at MAX IV Laboratory

**DOI:** 10.1107/S1600577520008723

**Published:** 2020-08-05

**Authors:** Thomas Ursby, Karl Åhnberg, Roberto Appio, Oskar Aurelius, Artur Barczyk, Antonio Bartalesi, Monika Bjelčić, Fredrik Bolmsten, Yngve Cerenius, R. Bruce Doak, Mikel Eguiraun, Thomas Eriksson, Ross J. Friel, Ishkhan Gorgisyan, Andrea Gross, Vahid Haghighat, Franz Hennies, Elmir Jagudin, Brian Norsk Jensen, Tobias Jeppsson, Marco Kloos, Julio Lidon-Simon, Gustavo M. A. de Lima, Robert Lizatovic, Magnus Lundin, Antonio Milan-Otero, Mirko Milas, Jie Nan, Alberto Nardella, Anders Rosborg, Anastasya Shilova, Robert L. Shoeman, Frank Siewert, Peter Sondhauss, Vladimir O. Talibov, Hamed Tarawneh, Johan Thånell, Marjolein Thunnissen, Johan Unge, Christopher Ward, Ana Gonzalez, Uwe Mueller

**Affiliations:** a MAX IV Laboratory, Lund University, PO Box 118, S-221 00 Lund, Sweden; b Department of Biomolecular Mechanisms, Max Planck Institute for Medical Research, Jahnstrasse 29, 69120 Heidelberg, Germany; c Helmholtz Zentrum Berlin für Materialien und Energie, Albert-Einstein-Strasse 15, DE-12489 Berlin, Germany

**Keywords:** beamline, macromolecular crystallography, MBA, micro-focus, serial crystallography, automation, remote operation

## Abstract

BioMAX is the first macromolecular crystallography beamline at the MAX IV Laboratory 3 GeV storage ring, which is the first operational multi-bend achromat storage ring. Due to the low-emittance storage ring, BioMAX has a parallel, high-intensity X-ray beam, even when focused down to 20 µm × 5 µm using the bendable focusing mirrors. The beam is tunable in the energy range 5–25 keV using the in-vacuum undulator and the horizontally deflecting double-crystal monochromator. BioMAX is equipped with an MD3 diffractometer, an ISARA high-capacity sample changer and an EIGER 16M hybrid pixel detector. Data collection at BioMAX is controlled using the newly developed *MXCuBE3* graphical user interface, and sample tracking is handled by ISPyB. The computing infrastructure includes data storage and processing both at MAX IV and the Lund University supercomputing center LUNARC. With state-of-the-art instrumentation, a high degree of automation, a user-friendly control system interface and remote operation, BioMAX provides an excellent facility for most macromolecular crystallography experiments. Serial crystallography using either a high-viscosity extruder injector or the MD3 as a fixed-target scanner is already implemented. The serial crystallography activities at MAX IV Laboratory will be further developed at the microfocus beamline MicroMAX, when it comes into operation in 2022. MicroMAX will have a 1 µm × 1 µm beam focus and a flux up to 10^15^ photons s^−1^ with main applications in serial crystallography, room-temperature structure determinations and time-resolved experiments.

## Abstract 2

BioMAX is the first macromolecular crystallography beamline at the first fourth-generation storage ring, the 3 GeV storage ring at MAX IV Laboratory. It is a highly automated, user-friendly, microfocus beamline that provides excellent performance for most macromolecular crystallography applications.

## 1. Introduction   

Macromolecular crystallography (MX) using synchrotron radiation has had an immense impact on structural biology (Duke & Johnson, 2010 ▸; Shi, 2014 ▸; Yamamoto *et al.*, 2017 ▸). Improved methods and instrumentation have played a pivotal role in the progress. The development of micro-crystallography using high-intensity, low-divergent X-ray beams has relied on better storage rings with improved insertion device technology. More accurate and reliable optics and diagnostics have made it possible to tailor and deliver these brilliant X-ray beams to the sample. The use of sample changers and better detectors has resulted in higher-quality data from smaller crystals, collected in less time. This has translated into rapid growth of scientific output thanks to improved analysis methods and rapid development of computing power for handling large amounts of data and its analysis. Important progress has also been made in sample production and crystallization making previous inaccessible studies possible, often using micro-crystals.

MAX IV Laboratory (MAX IV) in Lund, Sweden, operates the first multi-bend achromat (MBA) storage ring (Einfeld *et al.*, 2014 ▸), a technology providing lower-emittance electron beams resulting in more coherent X-ray beams with higher brilliance. Due to this improvement, these new MBA storage rings have been coined ‘fourth-generation storage-ring light sources’ (Borland *et al.*, 2014 ▸). The new MAX IV facility (MAX IV, 2010 ▸) was inaugurated in June 2016. MAX IV runs a 300 m-long linear accelerator, which is used as an electron source for a short pulse facility at its extension and top-up injections for two storage rings, with 1.5 GeV and 3 GeV electron energy, respectively. The 3 GeV MBA storage ring (Tavares *et al.*, 2014 ▸, 2018 ▸) has a circumference of 528 m with 20 straight sections. Its measured emittance of 0.3 nm rad agrees with the predicted performance. The facility has, presently, 16 funded beamlines of which ten are situated at the 3 GeV ring. The first beamlines to run commissioning experiments were BioMAX, the nano-science beamline NanoMAX (Johansson *et al.*, 2018 ▸) and the structural dynamics beamline FemtoMAX (Enquist *et al.*, 2018 ▸) at the short pulse facility.

The structural biology community is historically strong in Sweden with an early adoption of crystallography. The community benefitted relatively early from the locally available MX beamlines (Cerenius *et al.*, 2000 ▸; Mammen *et al.*, 2002 ▸; Ursby *et al.*, 2013 ▸) at the third-generation storage ring MAX II (Andersson *et al.*, 1994 ▸) starting with the first structure determined in 1998 (Unge *et al.*, 1998 ▸). The competitiveness of the MAX II MX beamlines declined as undulator-based beamlines at medium-energy storage rings became readily available elsewhere.

BioMAX is the first MX beamline at an MBA storage ring. The user operation started in March 2017 and it is now delivering a highly brilliant X-ray beam for regular users. With its state-of-the-art equipment and user facilities, its flexibility and microfocus performance, BioMAX is offering opportunities to utilize all established MX methodologies.

## 2. Beamline overview   

BioMAX was designed as a state-of-the-art macromolecular crystallography beamline in all respects with outstanding X-ray beam performance, thanks to the unique design of the 3 GeV storage ring. To reach this goal, we have relied to a large extent on available technology and hardware designs and improved these only when judged necessary. This has been made possible thanks to mature technology in many areas such as undulators, optical elements, mechanical designs for optics components, diffractometers, detectors and sample changers. BioMAX has a versatile, high-performant and automatic experiment setup. A large amount of work has been invested in developing an efficient and intuitive experiment control system and a computing environment that can handle the high data rates expected at the beamline. Table 1 ▸ summarizes the main parameters of the 3 GeV storage ring and the BioMAX beamline.

## 3. X-ray system   

**Figure 1:**
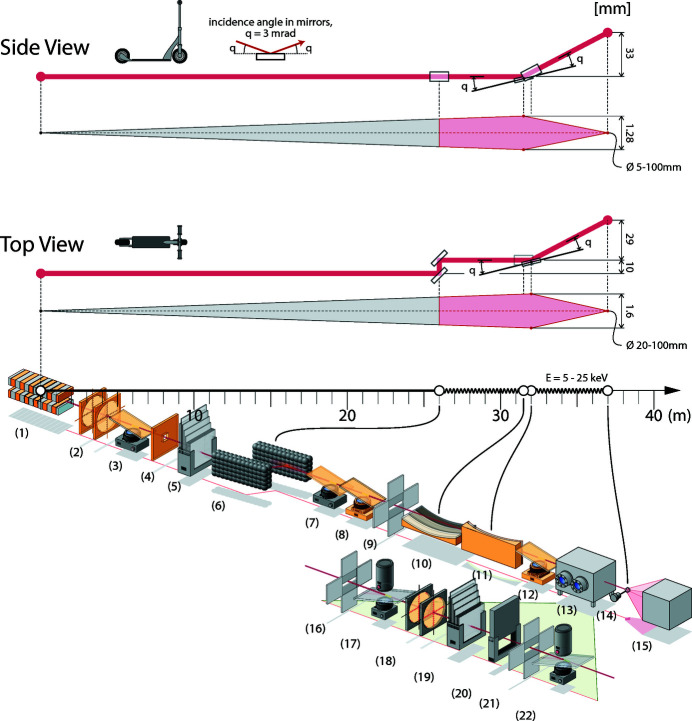
Beamline components from undulator to the detector: (1) undulator, (2) two front-end beam position monitors, (3) front-end fluorescent screen, (4) optics hutch fixed mask, (5) diamond filters, (6) double-crystal monochromator, (7) fluorescent screen, (8) NanoBPM, (9) slits, (10) vertical focusing mirror, (11) horizontal focusing mirror, (12) NanoBPM, (13) beam conditioning unit (BCU), (14) sample and (15) detector. The components in the BCU chamber are shown in the enlargement and include (16) slits, (17) beamviewer/intensity monitor, (18) two diamond beam position monitors, (19) attenuators, (20) shutter, (21) slits and (22) beamviewer/intensity monitor (illustration by J. Kvistholm).

The X-ray system has been designed to be as simple and reliable as possible while still giving high performance and flexibility covering a broad energy/wavelength range (5–25 keV/0.5–2.5 Å) and from a predicted focused beam size of 20 µm × 5 µm (FWHM, horizontal × vertical) to approximately a 100 µm-diameter beam (Thunnissen *et al.*, 2013 ▸). The beamline optics consists of a double-crystal monochromator and a pair of mirrors in Kirkpatrick–Baez (KB) geometry. The components from the undulator to the detector are shown in Fig. 1 ▸. The beamline vacuum system is windowless before the diamond window upstream of the diffractometer. The storage ring vacuum is protected by the small 1 mm × 1 mm opening of the fixed mask at the beginning of the optics hutch and the fast-closing valve in the front-end.

### 3.1. Undulator and front-end   

The BioMAX undulator (Hitachi Metals, Japan) is designed to give high brilliance in the energy range 5–25 keV. It is based on room-temperature, in-vacuum undulator technology with the use of hybrid structure (Yamamoto *et al.*, 1992 ▸). The undulator’s period length is 18 mm. It is 2 m long and has a minimum magnetic gap of 4.2 mm. It allows tapering to produce wiggler-like radiation (Tarawneh *et al.*, 2019 ▸). Table 2 ▸ summarizes the main parameters of the undulator.

**Figure 2:**
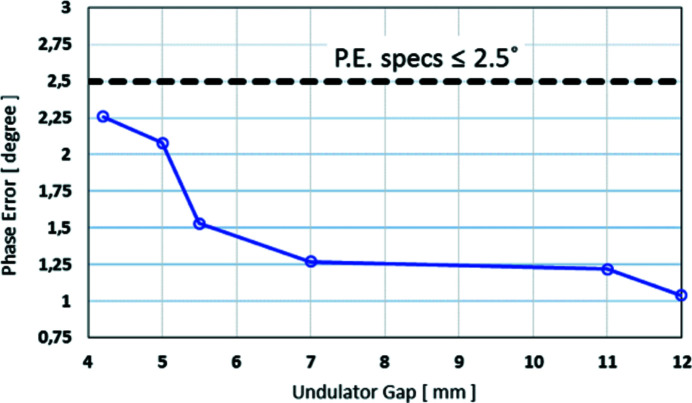
Measured phase error versus the magnetic gap of the BioMAX undulator.

The achieved effective *K* value is 2.19 at the minimum gap of 4.2 mm and the measured phase error (Fig. 2 ▸) is less than 2.5° for all operational gaps, as measured during the site acceptance tests carried out at MAX IV.

**Figure 3:**
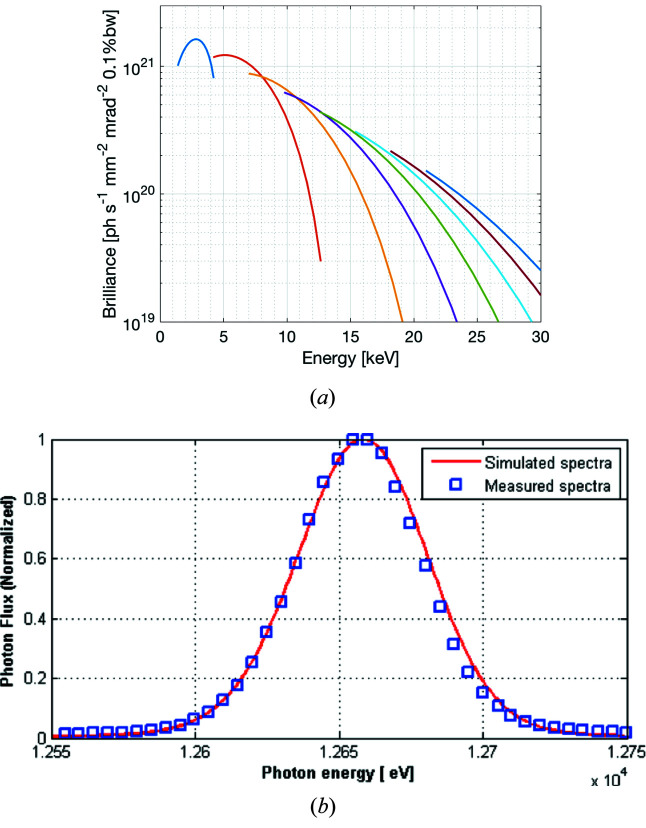
(*a*) Calculated brilliance of the odd harmonics 1–15 at 500 mA for *K* between 0.5 and 2.19. (*b*) Measured spectrum of the seventh harmonic at a fixed gap of 5 mm at 40 mA beam current with a simulated spectrum based on the measured magnetic field map.

The commissioning of the undulator started in spring 2016 with compensating the orbit distortion, due to the residual field integrals, by the dedicated corrector magnets in a feedforward scheme. Fig. 3 ▸ shows the estimated brilliance from the BioMAX undulator based on the baseline parameters of the MAX IV 3 GeV storage ring at 500 mA beam current (Tavares *et al.*, 2018 ▸). It also shows the seventh-harmonic peak recorded at 5 mm undulator gap with the corresponding simulated spectrum calculated from the measured magnetic field map of the undulator at 5 mm gap.

The front-end (FMB, Berlin, Germany) includes three fixed masks, two sets of blade-type X-ray beam position monitors, two moveable masks (forming one mask with horizontally and vertically adjustable offset and gap), one low-heat-load fluorescent screen, heat absorbers, safety shutters, a fast-closing valve with its triggering unit, an electron beam deflector and a bremsstrahlung collimator.

### 3.2. Beamline optical elements   

The BioMAX optics consisting of a horizontally deflecting double-crystal monochromator and a KB mirror pair is schematically represented in Fig. 1 ▸. A fixed mask with an opening angle of 50 µrad × 40 µrad (horizontal × vertical) is situated at the beginning of the optics hutch, matching the opening angle of the undulator beam (FWHM typically 25 µrad × 25 µrad horizontal × vertical calculated with *SPECTRA*; Tanaka & Kitamura, 2001 ▸). The highly parallel X-ray beam gives small beam footprints on the optics components. In particular, the small horizontal opening angle allows short deflecting elements also in the horizontal direction.

The monochromator is a horizontally deflecting Si(111) double-crystal monochromator (HDCM) (FMB Oxford, Oxford, UK) with 10 mm horizontal offset between the incoming and outgoing beams. The offset is kept constant giving a fixed beam position at the sample regardless of energy and beam focusing. Since the synchrotron light is predominantly horizontally polarized, the beam intensity is reduced for a horizontal deflection geometry compared with a vertical geometry. However, the effect at 12.4 keV is small (10%), only becoming significant at lower energies (around 50% reduction at 5 keV). A horizontal deflection geometry was chosen to increase the beam stability: if the two crystals would vibrate with respect to each other, the effect would be most pronounced in the scattering plane, in this case, the horizontal plane. Since the source is considerably larger in the horizontal plane (the electron beam σ is 50 µm in the horizontal versus 4 µm in the vertical), the relative effect would be smaller than if the scattering plane was vertical. This can be visualized by imagining the effect of a virtual movement of the photon source as the result of the first crystal vibration. A horizontal geometry has the additional advantage that the crystal holder can be made more rigid since weight is not as much an issue for a vertical rotation axis.

The Si crystals are symmetrically cut. The heat load on the first crystal is up to 124 W at 500 mA ring current (*K* = 2.19), with a peak power density on the monochromator crystal surface of 36 W mm^−2^ at 5 keV and 15 W mm^−2^ at 12 keV. This is normally reduced by using diamond filters upstream of the monochromator. Presently, in typical operation using a 25 µm diamond filter, undulator gap at 5 mm (seventh harmonic at 12.65 keV) and the storage ring operated at 250 mA, the power on the monochromator crystal is 41 W. The first crystal is side-cooled via liquid nitro­gen flowing through the Cu blocks holding the crystal, while the second crystal is side-cooled with Cu blocks that are cooled via Cu braids from the Cu blocks of the first crystal. To increase the heat transfer, In foils are inserted between the Si crystals and the Cu blocks. The liquid nitro­gen is supplied by a cryocooler XV (FMB Oxford, Oxford, UK). The first crystal has no adjustments while the second crystal is adjustable by stepper motors in pitch, roll and crystal-to-crystal distance. In addition, pitch and roll have piezo translations for fine adjustments. In this way, the first crystal that is in contact with the Cu blocks, through which the liquid nitro­gen is flowing, is firmly held, while the adjustable second crystal is cooled via Cu braids and therefore isolated from any liquid nitro­gen induced vibrations. The vibrational stability of the HDCM was measured (Kristiansen *et al.*, 2016 ▸) showing that the monochromator is indeed stable (25 nrad RMS relative pitch stability between the two crystals).

**Figure 4:**
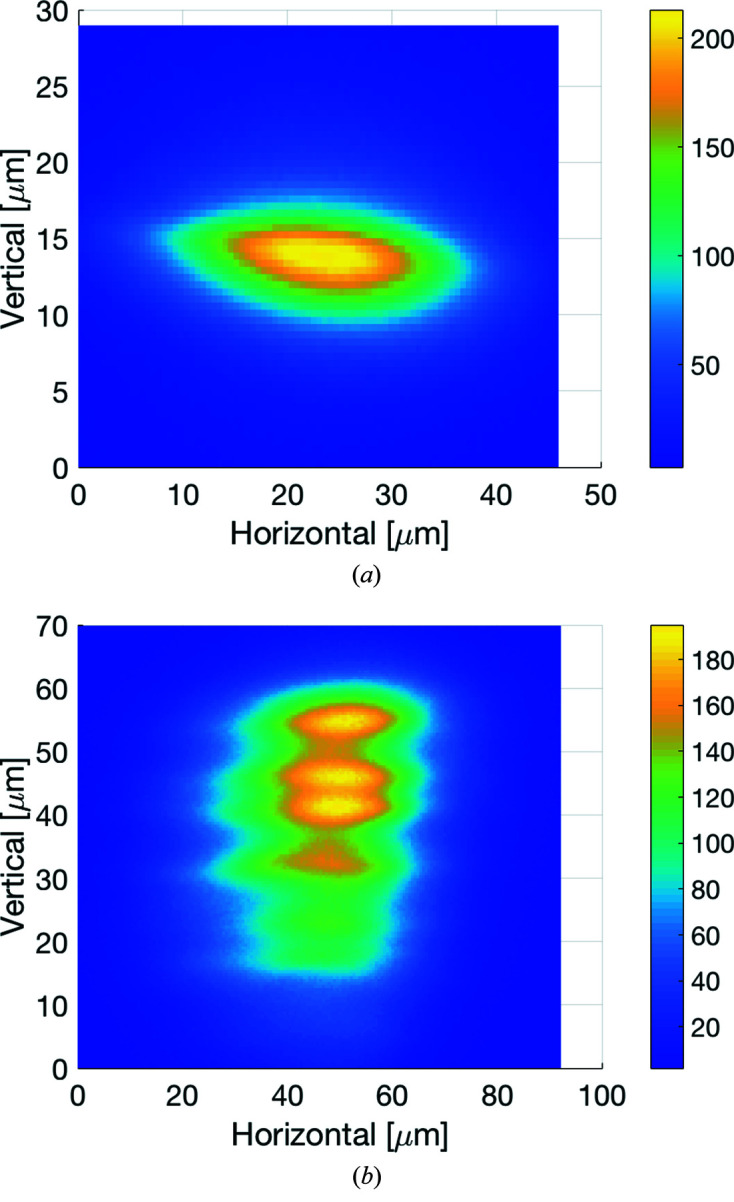
(*a*) Image of focused beam at the sample position at 13.5 keV. Actual beam size was measured with a knife-edge scan using the downstream slits in the BCU (350 mm upstream of the sample position) after optimizing the focusing at the slit position resulting in measured FWHM size of 22 µm × 4 µm (horizontal × vertical). Ray tracing in combination with thermoelastic finite-element analysis using *MASH* (Sondhauss, 2014 ▸) predicted a focus size of 20 µm × 3 µm. (*b*) Image of the defocused beam.

**Figure 5:**
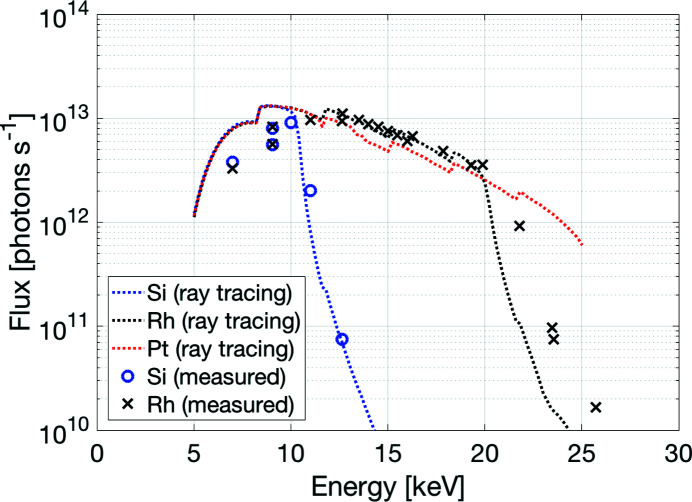
Calculated and measured flux at the sample as a function of energy at 200 mA for different mirror coatings (Si, Rh, Pt) without beam-defining aperture. The calculated flux curves are based on simulations using *MASH* (Sondhauss, 2014 ▸) and assumes an undulator specification of *K*_max_ = 1.92 while the measured flux curves used a conservative *K*_max_ of 1.8 (corresponding to 5 mm gap while the closed gap of 4.2 mm gives *K*_max_ = 2.19). The step increase at certain energies is due to the change of undulator harmonic and thus differs between calculated and measured curves. The flux curves drop rapidly above certain energies that corresponds to the mirror cut-off energies. In the ray-tracing the cut-off energy for the Rh coating is underestimated because the density is assumed to be 85% of the bulk density, whilst the actual density appears closer to 100%. The flux was measured using a PIN photodiode (AXUV100G, EQ Photonics GmbH) mounted on the detector 200 mm downstream of the sample position. Both calculated and measured flux are normalized to 200 mA storage ring current and includes the full flux at the sample (without any beam defining aperture or filters). The Pt-coated area was not measured and is not yet in normal user operation.

The X-ray beam is focused by two mirrors in KB geometry. The UHV vessel of the mirror system (FMB Oxford, Oxford, UK) houses the two silicon mirrors and their one-moment mechanical benders (Winlight X, France; see Table 3 ▸). Ray-tracing studies confirmed that each mirror only needs two degrees of freedom for final alignment and also the possibility to align the relative roll between the two mirrors. In addition, the mirrors need translations to change between the different coatings. The mirror system includes five degrees of freedom for the vertically focusing mirror (VFM) based on three small out-of-vacuum wedge-based vertical translations and two in-vacuum horizontal translations. The horizontally focusing mirror (HFM) support has three degrees of freedom with one large out-of-vacuum wedge-based vertical translation, one out-of-vacuum side translation and one in-vacuum cartwheel flexure rotation for adjustment of the mirror pitch. All degrees of freedom can be adjusted using stepper motors. In addition, the VFM and HFM mirror pitch angles have fine adjustment using piezo translators with 0.05/0.01 µrad resolution and 0.1/0.2 µrad repeatability for VFM/HFM. The VFM mirror substrate was pre-shaped to give an elliptic cylinder shape at the nominal bending position. Due to the small source size, only a modest demagnification is necessary, and the focusing mirrors are placed around 5 m upstream of the sample position. The measured focused beam size is 22 µm × 4 µm (FWHM, horizontal × vertical), see Fig. 4 ▸(*a*). To achieve a larger beam size, the beam is defocused by changing the bending radius of the mirrors to focus further downstream. Due to the small source size in the vertical direction, the out-of-focus beam shows striations caused by the slope errors of the vertical focusing mirror [Fig. 4 ▸(*b*)]. The predicted and measured flux at the sample for a storage ring current of 200 mA is shown in Fig. 5 ▸. Presently the ring is typically operated at 250 mA. The mirrors can also be translated out of the beam, and the experiment setup support adjusted so that the direct beam from the monochromator reaches the sample position. Both mirrors have three parallel optical areas with uncoated silicon for harmonic rejection at energies lower than 8 keV, Rh coating for use up to 20 keV and Pt coating for increased reflectivity at energies above 20 keV. The mirrors with benders were measured and characterized at the BESSY-II Optics Lab at HZB in Berlin. The performance of the bending system and the slope error was measured by means of the BESSY-NOM slope measuring profiler (Siewert *et al.*, 2004 ▸) which allows an inspection of the optics for the spatial frequency range between 1.2 mm up to full aperture length (Siewert *et al.*, 2016 ▸). A white-light interferometer (WLI) was used to measure the micro-roughness of the mirrors giving an expression for the higher-spatial frequency error which would cause loss of photons because of scatter. The optics hutch components also include three diagnostic modules with filters, slits and beam monitors (FMB Oxford, Oxford, UK) and monochromatic safety shutters (Axilon, Germany).

### 3.3. Beam conditioning unit   

The beam conditioning unit (BCU) was developed and built by the instrumentation group at EMBL-Hamburg based on an earlier design (Cianci *et al.*, 2017 ▸) in collaboration with MAX IV. The BCU vacuum chamber is 690 mm flange-to-flange along the beam and maintained at high vacuum (typically <5 × 10^−8^ mbar). The unit includes two sets of horizontal and vertical tungsten slits that define two apertures. Downstream of each pair of slits is a rotational device with four positions that can be moved into the beam. Two of the positions are occupied by diagnostic tools: a YAG-scintillator screen that can be viewed with a camera, and 1 µm titanium foil at 45° to the X-ray beam, which scatters photons for detection using the PIN diode intensity monitor directly above the foil, while the other two positions are presently not used. Downstream of the first slits/diagnostic set, there are two B9 diamond X-ray beam positioning monitors (XBPM) (CIVIDEC Instrumentation GmbH, Vienna, Austria) based on a 50 µm-thick single-crystal diamond foil (3 mm-diameter active area) with a 100 nm titanium coating forming four quadrants. Downstream of the XBPMs there are three attenuator wheels, each with ten positions containing a range of metal foils and one through (no foil) position. The setup provides 1000 combinations of attenuators allowing for rapid and precise changes of the beam intensity. Following the attenuators is a fast piezoelectric X-ray shutter (CEDRAT Technologies, Meylan, France) to control beam delivery to the sample. The slits and the XBPM *xy*-supports are moved using attocube ECS3050 piezo positioners (attocube systems AG, Munich, Germany) with nanometre resolution.

### 3.4. Beam diagnostics and stability   

**Figure 6:**
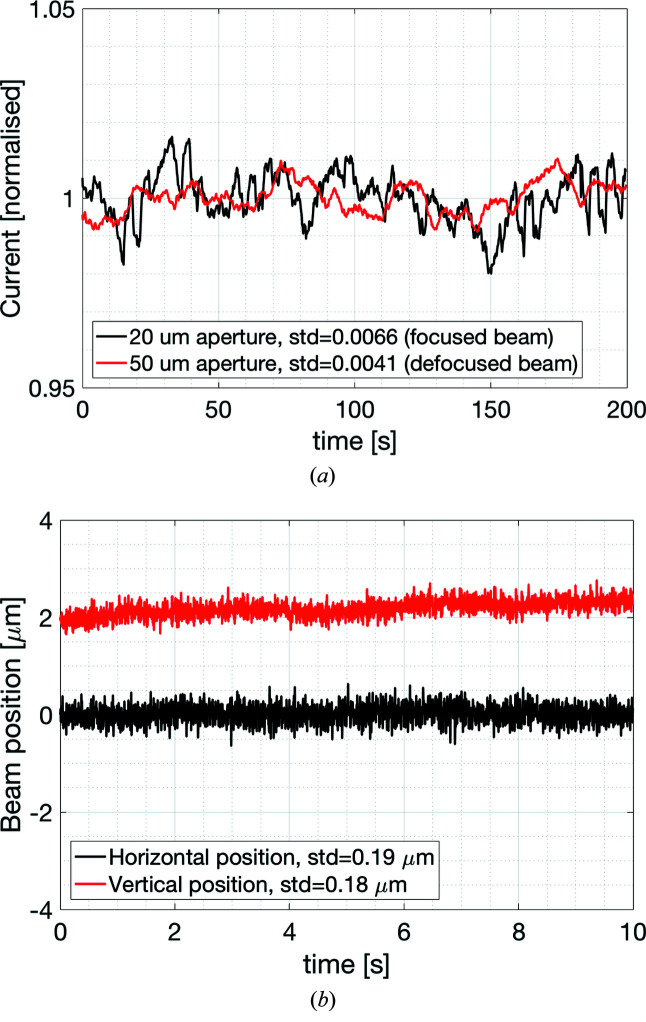
(*a*) Beam intensity at the sample position through the 20 µm-diameter aperture with the focused beam (black) and 50 µm-diameter aperture with a defocused beam (red) (FWHM size around 40 µm × 60 µm, horizontal × vertical). The RMS intensity variation is 0.7% and 0.4%, respectively. Measurements made with both optics feedback loops in operation. Data collected at 5 Hz using the diode mounted on the detector cover. (*b*) Position measured at 200 Hz using the XBPM in the BCU with the feedback loop to the KB mirrors turned off. The BPM is about 0.5 m upstream of the sample position but 4.5 m downstream of the mirrors giving a good measure of the beam stability at the sample position. The vertical position is offset by 2 µm in the figure for clarity. The measured RMS deviation from the mean is about 0.2 µm in both horizontal and vertical direction. The beam without feedback is not always this stable on the seconds time scale, which is the reason for the mirror feedback loop giving the long-term stability shown in (*a*).

A stable beam and diffraction signal require diagnostics that can detect beam movement and, if needed, be used for beam position feedback. Diagnostics are also needed to measure beam shape and position during alignment and operation. The electron beam in the storage ring is controlled by beam position monitors upstream and downstream of the BioMAX undulator. The front-end includes two *xy* blade-type beam position monitors. A semi-transparent diamond fluorescent screen (0.4 mm thickness at 45° to beam trajectory) can be inserted in the beam at the beginning of the optics hutch, before the optical elements, to verify the position of the incoming beam. A white beamstop located after the monochromator is covered in P43 (Gd_2_O_2_S:Tb) allowing visualization of the direct white beam by moving out the first monochromator crystal. A non-transparent fluorescent screen (75 µm-thick yttrium oxide) can be inserted after the monochromator and is viewed by the same camera that is used for the white beamstop. The optics hutch is also equipped with two NanoBPMs (FMB Oxford, Oxford, UK; Silfhout *et al.*, 2011 ▸), one before and one after the KB mirrors. These consist of a 125 µm-thick Kapton foil that scatters the beam through a pinhole aperture and onto a fluorescent screen where a CMOS sensor detects the visible light. The signal from the NanoBPM, located between the monochromator and the KB mirrors, is routinely processed and used for a 10 Hz feedback loop acting on the second monochromator crystal pitch-and-roll piezo motors to correct for a slow drift of the beam or during changes of energy. In the experimental hutch, there are two diamond beam position monitors in the beam conditioning unit (see §3.3). The first of these beam position monitors is routinely used in a 10 Hz feedback loop to the piezo adjustments of the pitch of the mirrors to correct for long-term drifts and to automate beam positioning after energy changes (see Fig. 6 ▸). The BCU also includes two fluorescent screens for visual inspection of the beam close to the sample position. After the BCU vacuum window, as part of the micro-diffractometer assembly, there is a BGO (bis­muth germanate) fluorescent screen that can be inserted at the sample position. Finally, a Si PIN photodiode mounted on the area detector protective cover is used to measure the beam flux (see Fig. 5 ▸).

## 4. Experiment endstation   

**Figure 7:**
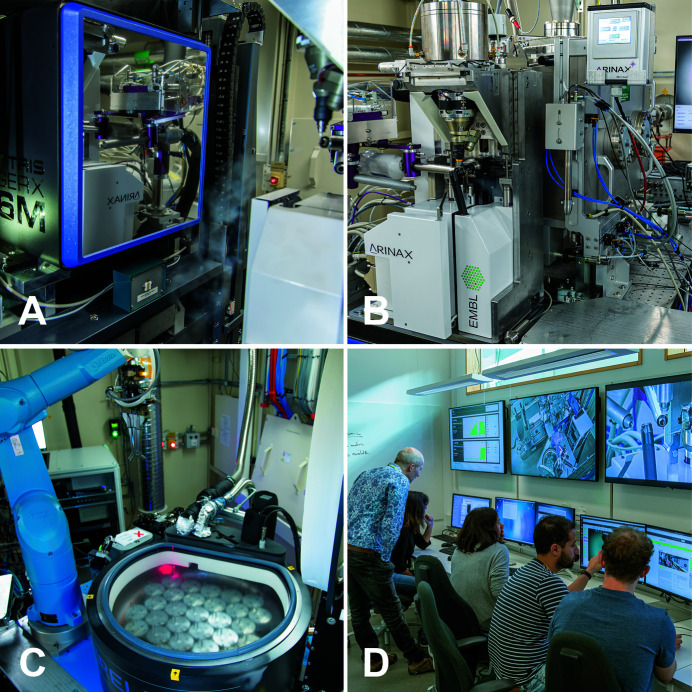
Experiment setup: (A) Eiger 16M detector mounted at the detector table during data acquisition. (B) MD3 with Mini-kappa, XRF detector as well as Cryojet and HC-Lab nozzles mounted on the REX nozzle exchanger. (C) ISARA sample changer with double universal puck gripper and laser puck identification. (D) The control room at BioMAX during user operation.

The main elements of the experiment station setup are the diffractometer, the area detector and the sample changer (Fig. 7 ▸). The diffractometer is mounted on a five-axis support table (Research Instruments, Germany) that also carries a REX rapid nozzle exchanger (Arinax, France) for the sample cryo nozzle and the humidity controller nozzle, the fluorescent detector holder, the XR-100SDD fluorescence detector (Amptek, USA) and the beam conditioning unit.

### 4.1. Micro-diffractometer   

The goniometry is performed by an MD3 micro-diffractometer (Arinax, France). It is equipped with a high-resolution on-axis zoom-microscope (OAV) with focused back-light and front-light LED illumination. The OAV can be used for crystal centering and, when the BGO (bis­muth germanate) screen is translated to the sample position, to assist with beam alignment. The omega axis is an air-bearing rotation stage, which is pointing downwards to minimize the gravitation-related sphere-of-confusion during rotation. The axis can be operated up to a speed of 800° s^−1^. The sphere-of-confusion has been measured with the OAV by observing the tip of a thin, conical glass needle to be 120 nm radius at a speed of 100° s^−1^ using the standard omega-goniometer head. A nanometre precision translation table is mounted on the omega axis for fine positioning of the crystal in the *x*- and *y*-direction, perpendicular to the axis. The omega axis itself sits on a *z*-axis translation stage. The combination of the very precise crystal alignment and the fast omega-axis allows one to perform helical scans, which are omega rotations with a synchronized *xyz*-motion creating a trajectory, for example along a needle-shaped crystal. This feature is regularly used to distribute the absorbed X-ray dose over the whole crystal volume when a suitable translation, roughly parallel to the omega axis, can be defined (Zeldin *et al.*, 2013 ▸). The omega axis can be equipped with a mini-kappa goniometer MK3 (Arinax, France), which increases the rotational freedom to phi and kappa at the expense of some degradation of the sphere-of-confusion (from 120 to 450 nm). A smart and switchable sample magnet senses the presence of a crystal sample holder. Another alternative is the mounting of a crystallization plate translation stage. This stage can be used for *in situ* screening purposes. The MD3 is supporting a penta-aperture for fast beam shaping with circular apertures of 5, 10, 20, 50 and 100 µm-diameter. Downstream, a 22 mm-long molybdenum beam-cleaning aperture with 400 µm inner diameter and a 200 µm cleaning aperture at the end eliminates upstream scattering contamination. Finally, the MD3 has a movable 300 µm-wide beamstop. The MD3 is also equipped with a Colibri fast shutter (Arinax, France).

The temperature around the mounted crystal is controlled by a Cryojet XL (Oxford Instruments, UK). The normal sample temperature is 100 K. In addition, a room-temperature humidity controller HC-lab (Arinax, France) is available for dehydration studies and as a room-temperature environment, both for single-crystal data collection and for serial crystallography raster scans. Both gas nozzles are mounted on the remotely controlled nozzle exchanger, which can perform environment changes within less than a second.

Recently, the MD3 has been upgraded with a Power-PMAC motion controller (Delta Tau, CA, USA), which allows the running of fast synchrotron-based serial crystallography (SSX) raster scans over an area of 3 mm × 3 mm, and scanning of approximately 60 data points per second within a snake motion trajectory. This option allows the collection of a complete serial crystallography data set from a small multi-crystal holding chip in minutes.

### 4.2. Area detector   

The area detector is an EIGER 16M (Dectris, Switzerland) hybrid pixel detector with 75 µm pixel size and 311 mm × 328 mm sensitive area (of which 6.6% is inactive area between modules). The sensor is made of 450 µm-thick silicon. It can collect full-frame data with a frequency of up to 133 Hz. In 4M pixel region-of-interest scanning mode, using only the central eight modules, it can be operated at up to 750 Hz. The detector is mounted on a granite platform with a four-axis support including motorized control of detector distance, two vertical translations giving up to 10° 2Θ-rotation and a 40 mm horizontal translation, which is used when switching to collecting diffraction data without the KB mirror optics. The detector distance from the sample position can be adjusted in the range 146–1000 mm.

### 4.3. Sample changer   

The ISARA sample changer (Irelec, France) consists of a Stäubli TX-60 six-axis industrial robot, the control unit and a large sample Dewar that supports 29 universal pucks (Unipucks) for the storage of up to 464 samples. The decision was made to support only samples mounted in Unipucks to eliminate the need for commissioning and regular maintenance of two sets of very diverse mounting tools. The sample changer exchanges hardware signals with the MD3 via potential-free contacts, to synchronize the sample mount operation. The detector table and MD3 controlled components are interlocked with the sample changer to prevent collisions – the sample changer waits for the go-ahead from the MD3 before entering the sample area, and while in the sample area the sample changer prevents the MD3 and the detector table from moving. The sample changer has been in user operation since October 2018, operating very reliably; total sample loss failure rate <0.05%. A standard sample exchange takes approximately 25 s. Typically, users mount between 80 and 96 samples in 8 h. We have equipped the sample changer with an in-house-developed Unipuck interface location system (ISARAloader), which guides the user or staff during loading and unloading operations, to prevent any misplacement of the pucks within the 29 positions of the Dewar. ISARAloader consists of a class 2 laser indicating the selected puck position, a pair of digital servo motors (Corona DS238MG) in a 3D-printed holder, allowing each motor to rotate 150° orthogonally to cover the whole Dewar area, and an Arduino Uno (https://www.arduino.cc) controlling the device. The controlling software is built using a Python Django server running on a Raspberry Pi 3B single board computer (https://www.raspberrypi.org). The server authenticates the users through the MAX IV DUO system (https://duo.maxiv.lu.se), and prepares a position in the Dewar by polling information about available puck positions from a Tango server and information about available pucks from the ISPyB database (see §5). An available position is then assigned to the puck selected through the touchscreen interface written in HTML5 and JavaScript, moving the motors and pointing with the laser to where the puck should be placed. Once the puck is detected by the sample changer, the information is stored in the ISPyB database and is promptly available for user operation. Users can load and unload the Dewar independently after initial training. Typical loading and unloading times are approximately 15 min for 10 pucks holding up to 160 samples. The sample changer is calibrated every week before the start of the user operation, via a semi-automatic teaching procedure. The sample changer is usually warmed up every week to remove ice building up during the operation. With this maintenance, icing is not a problem.

### 4.4. High-viscosity extruder injector   

In order to establish and use new experimental techniques relevant for the MicroMAX beamline, we started a collaboration with Bruce Doak from the Max Planck Institute for Medical Research, Heidelberg, Germany, to develop a high-viscosity extrusion (HVE) injector for SSX applications at BioMAX (Shilova *et al.*, 2020 ▸; Andersson *et al.*, 2019 ▸). This device can be easily mounted on the MD3 omega axis (instead of the single axis or mini-kappa) and uses the MD3 alignment translations and sample environment including the OAV. It can extrude microcrystals embedded in an appropriate high-viscosity matrix, such as grease, LCP or other matrices (Kovácsová *et al.*, 2017 ▸), through a glass capillary (typically with an inner diameter of 75–100 µm). The extrusion speed is 1–4 mm s^−1^, which translates to a beam interaction time of a few milliseconds for 5–10 µm large crystals using the focused X-ray beam. This setup is complemented by a Shimadzu HPLC-pump creating hydraulic pressure acting on a piston with an 8× amplification, and a He pressure controller creating a sheath He-gas stream around the extruder capillary. The extruder control is fully remote.

## 5. Experiment control and data evaluation   

**Figure 8:**
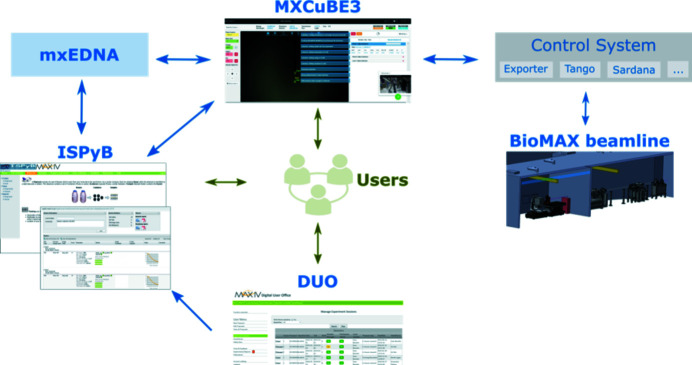
Overview of control and analysis software at BioMAX. Arrows in green show the interactions between users and software, while arrows in blue show the communication between different software or system.

The experimental setup at BioMAX is highly automated and enables multiple cutting-edge experimental possibilities. To meet the resulting high-performance requirements and to help users to manage the large amount of sample information and experimental data produced, but also to ease the user experience for complex experiments, different control and analysis software packages are implemented and actively developed. These are *MXCuBE3* (Mueller *et al.*, 2017 ▸), ISPyB (Delagenière *et al.*, 2011 ▸), *EDNA-MX* (Incardona *et al.*, 2009 ▸; https://github.com/olofsvensson/edna-mx) and various lower-level control systems (see Fig. 8 ▸). Users only need to interact with ISPyB and *MXCuBE3* directly during the experiment. In brief, after users schedule their beam time session with the MAX IV DUO system they upload their sample information as a spreadsheet to ISPyB. During the beam time, *MXCuBE3* imports the proposal, user and sample information from ISPyB. It also exports experiment details to ISPyB, including beamline and data collection parameters. *MXCuBE3* automatically triggers *EDNA-MX* for data analysis or auto-processing, depending on the experiment type. The results are fed back to *MXCuBE3* and ISPyB, where the experiment information and data processing results can be accessed by the users both during and after their beam time.

The BioMAX control system (Eguiraun *et al.*, 2017 ▸) is based on Tango (Chaize *et al.*, 1999 ▸) and Sardana (Coutinho *et al.*, 2011 ▸). Tango is a free, open-source distributed control system in use and active development in most of the European synchrotrons. Sardana is a software environment on top of Tango providing tools to be able to, for example, steer motors, define and act on pseudo-motors, acquire signals and run macros. Tango and Sardana as well as the motion controller Icepap (Janvier *et al.*, 2013 ▸) are adopted as MAX IV standards. At BioMAX the beamline motors are steered through this controller except for the diffractometer and sample changer, which are integrated systems with dedicated equipment. There are several easily configurable user interfaces based on Taurus (http://taurus-scada.org/) that display motor positions and sensor information. The beamline synoptic displays a graphical representation of the physical beamline structure enabling an intuitive way of interaction with the control system.

### 5.1. *MXCuBE3*   

**Figure 9:**
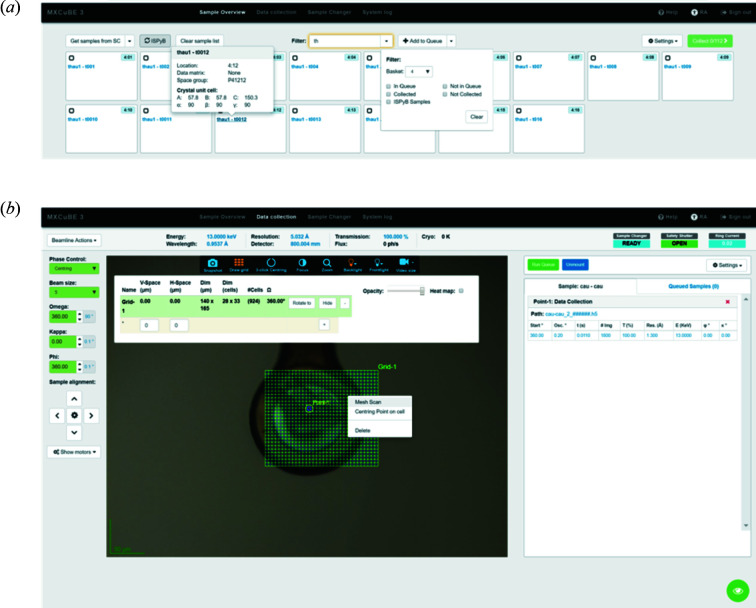
*MXCuBE3* user interface. (*a*) Sample view. (*b*) Data collection view.

*MXCuBE3* is the central beamline control software for users to carry out their crystallography experiments, either on-site or remotely. The MXCuBE project (Macromolecular Xtallography Customized Beamline Environment) (Gabadinho *et al.*, 2010 ▸; Oscarsson *et al.*, 2019 ▸) is supported by most synchrotron facilities in Europe. *MXCuBE3* is, currently, the latest version, which takes advantage of web technology and was designed to be intuitive and user-friendly (see Fig. 9 ▸). *MXCuBE3* was developed by MAX IV and ESRF, and first deployed at BioMAX at the beginning of its user operation in 2017. There are two main interfaces in *MXCuBE3*, the sample view [see Fig. 9 ▸(*a*)] and the data collection view [see Fig. 9 ▸(*b*)]. In the sample view, each sample is represented by a card, which contains all relevant information of the individual specimen, including sample and protein name, position in the sample changer Dewar and associated experimental status and results. Users can have a quick and easy overview of the status of all samples, but also inspect an individual sample for details. The view provides filter functions so that users can focus on a small group of samples by puck position, name, experimental status *etc*. The users can either mount the samples one by one or select several samples (a ‘queue’) to mount sequentially in batch mode. It is possible to define the data collection parameters in advance for queued samples, and there are provisions to import data collection strategies from ISPyB.

The data collection view allows users to control the beamline and run the experiment. A status bar at the top shows beam parameters relevant to the experiment (energy, transmission, flux), the detector position and the resolution at the detector, the sample changer and safety shutter status, and the storage ring current. The beamline actions menu at the top left gives users access to a list of scripted functions to, for example, start beam delivery (*e.g.* after a storage ring injection), check the beam stability, and to move the detector and robot out of the way before going into the experimental hutch. The center of the window displays the interactive live sample video feed from the MD3 camera. Users can center and align the sample by three-click centering, and define points, lines and meshes on the sample. A right-click on the defined object(s) brings up a list of all possible associated actions, including experimental procedures, such as ‘Standard Data Collection’, ‘Characterization’, ‘Helical scan’, ‘Mesh scan’, ‘XRF’ and ‘Energy Scan’. After choosing one of these procedures, a pop-up window shows the relevant input parameters that the users can edit. The data collection can then be executed immediately or stored in the sample queue. To the left of the sample video, there is a frame showing the MD3 motors defining the sample position, orientation and beam collimator size. To the right side, the users can toggle between the list of collection tasks defined for the currently mounted sample and, if a sample queue is defined, the list of samples. The level of automation of the experiment is controlled with the ‘Settings’ drop-down menu. For example, the ‘Loop and uCrystals IDentification’ (LUCID; Francois *et al.*, 2014 ▸) algorithm can be launched automatically after sample mounting with the robot (usually this is the default) and snapshots of the crystals can be taken during data collection. The communication between *MXCuBE3* and beamline devices and motors are via Tango or Sardana, except for MD3, which uses the exporter interface provided by the vendor.

### 5.2. ISPyB/EXI   

ISPyB (Information System for Protein Crystallography Beamlines; Delagenière *et al.*, 2011 ▸) is a laboratory information management system combining sample tracking and experiment reporting at synchrotron beamlines. Like the MXCuBE project, it is supported by the major synchrotron facilities in Europe. ISPyB was adapted to the BioMAX environment, particularly in support of sample shipment handling and the DUO user portal. The web-based interface offers an easy way for the users to access summarized information about all the data collections made during all the beam time sessions across different proposals, and also to create and edit experiment reports. Users can also examine the diffraction images (as thumbnails), crystal snapshots, collection parameters and beamline information, as well as examine and download the results from autoprocessing pipelines. EXI (https://exi.esrf.fr) is the new interface for ISPyB, which was mainly developed by ESRF with the aims of faster display, easier use and more intuitive user-interface. EXI is presently in commissioning at BioMAX.

### 5.3. Characterization and automatic data processing   

When a dataset is collected in *MXCuBE3*, automatic data analysis or data reduction pipelines are triggered and managed in EDNA (Incardona *et al.*, 2009 ▸), a framework for plugin-based applications for online data analysis at synchrotrons. The MX part (EDNA-MX; https://github.com/olofsvensson/edna-mx) is still active and part of the MXCuBE collaboration.

Upon data collection, three automatic reduction pipelines are currently triggered at BioMAX: fastdp (Winter & McAuley, 2011 ▸), EDNAproc (Incardona *et al.*, 2009 ▸) and autoPROC (Vonrhein *et al.*, 2011 ▸). EDNA uses execution plugins to handle the input and output from the different pipelines in a generic way. The existing plugins for EDNAproc and autoPROC were modified to adapt to BioMAX computation and ISPyB environment, while the execution and control plugins for fastdp were developed from scratch. At the end of a fastdp run the fast phasing pipeline fastep (https://github.com/DiamondLightSource/fast_ep) is launched if an anomalous signal is detected in the data. Adaptation of ISPyB to display the results from fastep is underway. A strategy calculation aiming to take full advantage of the EIGER 16M detector capabilities, based on a low dose per image combined with high data multiplicity, is also under development.

All automatic processing is executed on the MAX IV online high-performance computing (HPC) cluster. To take full advantage of the HPC resources and speed up the processing, *XDS* (Kabsch, 2010 ▸) was modified to run on multiple nodes in COLSPOT and INTEGRATE steps. Both fastdp and EDNAproc are fast and provide immediate feedback within a few minutes. It usually takes a little longer for autoPROC jobs to finish, but it performs more complex analysis, problem detection and also anisotropic analysis using STARANISO (Tickle *et al.*, 2018 ▸), complementary to the other two pipelines.

## 6. Computing infrastructure   

**Figure 10:**
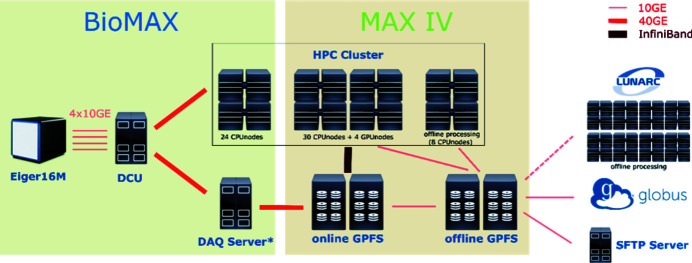
Scheme of the IT infrastructure for data processing.

The data acquired by the EIGER 16M detector is transferred to the Detector Control Unit (DCU) where it is compressed and transferred with 40GE optical fibers to the data acquisition server (DAQ) and written to the high-performance online storage via GPFS (see Fig. 10 ▸). The online storage is mounted to the online HPC cluster by GPFS over InfiniBand, which is ideal for the high I/O demanding reduction pipeline. In addition, the DCU is directly connected to the online HPC cluster for on-the-fly analysis, which is critical for X-ray centering and SSX experiments. Both the online storage and HPC cluster are centralized IT-resources at MAX IV and are shared among beamlines for operation. Currently the online HPC has 4 GPU nodes and 54 CPU nodes (Intel Xeon CPU E5-2650 v4 @ 2.20 GHz, 128 GB RAM) with 24 physical cores per node. There are 24 CPU nodes with larger RAM (256 GB) which are prioritized for BioMAX data analysis jobs.

Data on the online storage are synchronized to an offline storage where it is available for users who wish to access their data after their beam time. Users can download their data from the offline storage, by Globus Connect (Foster, 2011 ▸; Allen *et al.*, 2012 ▸) or SFTP, but not directly at the beamline. The data lifetime on the offline storage is anticipated to be 3–6 months. Afterwards all experimental data will be archived on a tape storage system (under development). For offline processing after beam time, users can use the MAX IV offline processing cluster, which currently has 8 cluster nodes (Intel Xeon CPU E5-2650 v3 @ 2.30 GHz) with 20 physical cores per node. MAX IV users can also apply to use the Supercomputing Center at Lund University (LUNARC) for data processing.

Both the online and offline processing HPC clusters have the same operating system and software setup and also share the same home folder. Users can either access the cluster by SSH (only available from the beamline) or login to the HPC Desktop via the Cendio Thinlinc client (https://www.cendio.com/thinlinc/). Slurm (https://slurm.schedmd.com/) is used for job scheduling. In general, the interactive MX software is installed and available using the PReSTO project (see https://www.nsc.liu.se/support/presto/, where there is also a comprehensive list of available data processing and phasing software). PReSTO is an EasyBuild distribution for cluster environments, which facilitates tuning, handling and compatibility issues typically encountered for software in a cluster setting.

## 7. Beamline support facilities   

**Figure 11:**
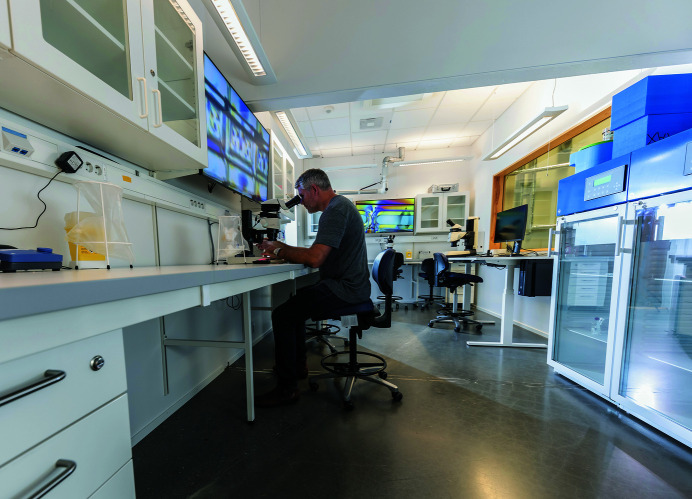
The BioMAX sample preparation laboratory

The BioMAX beamline has a sample preparation laboratory (see Fig. 11 ▸) in direct connection to the experiment setup. Here, users can handle cryo-cooled samples, mount samples and cryo-cool these prior to the beam time. There are two high-end Leica M80 stereo microscopy stations with video screens for sample inspection and mounting, and a higher magnifying microscope, Olympus BX53, for the characterization of micro-crystals for SSX. There are 20°C and 5°C crystal storage cabinets. The BioMAX sample preparation lab is developed to be complementary to the MAX IV Biolab as well as the future MicroMAX sample lab and biophysical laboratory. All lab facilities are located within a 20 m range.

The MAX IV Biolab is the central laboratory facility for the support of life-science-related synchrotron experiments carried out at various beamlines. The lab complements the beamline-attached sample preparation facilities and is being developed to offer extended instrumentation access and support. Currently the Biolab has the capability of supporting the users with creating solutions, concentration and centrifugation of proteins, dialysis, gel electrophoresis, UV-Vis spectroscopy with small sample volumes, dynamic light scattering (DLS), microscope, storage of chemicals and samples at 4°C, −20°C and −80°C, anaerobic work in a glovebox, and consumables for their experiments in the Biolab. The lab is also equipped with an HPLC (high-performance liquid chromatography) with a fractionator in a thermostat and a separate set of columns enabling protein purification capability.

## 8. Facility access   

Access to the BioMAX beamline can be obtained through the MAX IV proposal system within the digital user office portal DUO based on the software developed at the PSI (Paul Scherrer Institute, Switzerland). The DUO deployed at MAX IV has been adapted and extended to cover the needs of MAX IV and includes proposal, review and scheduling tools, a user and publication database, as well as tools for safety and feasibility review.

Submitted proposals are reviewed by beamline staff for feasibility and are subsequently sent to an independent Programme Advisory Committee within structural biology for scientific review. In parallel the safety team at MAX IV performs a safety review. Based on the result from these reviews, the MAX IV management takes the final decision on beam-time allocation. The modes of access that are provided are normal proposals (single project) and Block Allocation Group (BAG) proposals in order to enhance flexibility for the users and more efficient use of beam time. Training sessions for BAG proposal members are offered periodically.

### 8.1. Remote operation   

Automation of sample exchange and other experiment tasks means that users seldom need to go inside the experiment hutch during their experiment. This facilitates the implementation of remote experiments. Remote access to synchrotron facilities is often a key requirement to attract non-local and industry-based MX users, and it allows, in principle, more efficient use of the beam time; therefore, it has been a high-priority feature at BioMAX. The first fully remote experiment took place in October 2019. Currently, any user who has collected data in person at MAX IV at least once per year or has attended BAG training can request remote access.

Remote users at BioMAX have total control of their experiment and they have access to the same experimental resources as local users, except where manual sample handling is required, or non-standard instrumentation setups are being used. Experiments at room temperature, injector-based serial crystallography or collection from hazardous samples are not suitable for remote access. Samples for remote access must be mounted on standard SPINE pins and loaded in Unipucks.

Users can access the beamline computers from external locations via a VPN connection and then log in to designated remote servers via the Thinlinc client remote desktop. The MAX IV VPN server supports two-step authentication and dual routing so that the user computer remains in its own local network and only the Thinlinc remote desktop is in the MAX IV network. For data processing, users can connect remotely any time to the HPC offline cluster. For data collection, remote access is restricted to the duration of the beam-time session.

## 9. Beamline use and results   

### 9.1. BioMAX use and user statistics   

BioMAX has mostly been used for macromolecular crystallography with both academic and industrial users. Single-axis data collection at cryogenic temperatures is the most common experiment setup requested by users. The fast readout of the detector and high flux makes it possible to collect 360° data in little more than half a minute, using a standard 0.1° rotation and an exposure of 10 ms per frame. One of the top priorities at BioMAX has been to implement automation to minimize the time spent mounting the sample, centering in the beam and evaluating the crystal diffraction. Use of the sample changer reduces the mounting and dismounting time per sample to less than 25 s. When the X-ray beam is fully focused (20 µm × 5 µm FWHM) it is possible to obtain a high-flux, stable 6 µm × 5 µm beam at the sample position by inserting a 5 µm-diameter aperture 32 mm upstream of the sample position, which allows the study of microcrystals. The use of the small-sized beam in conjunction with helical data collection optimizes conditions for needle-shaped crystals. For larger crystals, the standard practice is to defocus the beam to a size of 50 µm × 50 µm FWHM, with a choice of 50 µm- or 20 µm-diameter defining apertures. Despite the presence of vertical structure in the unfocused beam arising from the focusing mirror imperfections, test data sets show excellent quality. Beam defocusing also creates a practically parallel beam, which, in combination with the low point spread function of the EIGER detector, can be used to resolve unit cells larger than 1000 Å at high resolution. Although the beamline total energy range is 5–25 keV, the standard range offered to users currently is 6–19.6 keV. This range covers many absorption edges of interest in biological samples. Using the lower energy part of the range also allows the measurement of significant signal from heavy metals or even native elements without absorption edges in the range. At the higher part of the energy range around 19.6 keV, it is possible to measure a complete data set to 0.8 Å resolution, of interest for small organic molecule work, at the expense of lower detector efficiency.

So far, until the end of 2019, BioMAX has received 314 individual users making 640 visits in total. In 2019, 199 users made 337 visits in total. Altogether, 10248 datasets (7561 during 2019) have been collected while examining 13480 samples (SPINE sample holders), 9658 of these were in 2019 when the sample changer was in operation. Serial crystallography data collections are not included in this dataset/sample statistics. BioMAX has had eight different industrial users paying for their beam time with several additional new inquiries during spring 2020, illustrating the importance of quick access for industrial partners. In addition, there are industrial partners that have received funding for beam time from external funding agencies for different projects.

During spring 2020 BioMAX has remained fully operational switching to nearly complete remote operation which has minimized the number of users having to cancel their beam time. Fast access applications for SARS-CoV-2 research has also been implemented.

### 9.2. Results   

**Figure 12:**
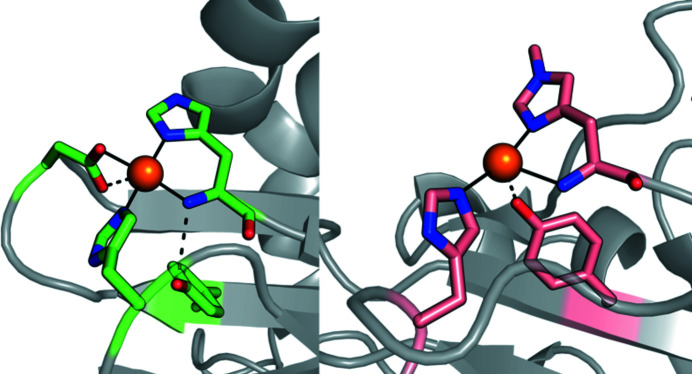
Copper binding sites of two different proteins. Left: LaX325 protein belonging to the newly identified LPMO-like protein family X325. Right: cellulose cleaving LPMO enzyme TaAA9.

This section highlights some of the studies that have been performed at BioMAX. Labourel *et al.* (2020 ▸) characterized a new family of proteins, named X325, which can be found in various fungal lineages. The X325 structure was determined in three different space groups including two structures determined using data collected at BioMAX (in 2018) to 2.1 Å and 1.8 Å resolution. The three-dimensional structure of X325 revealed an overall lytic polysaccharide monooxygenases (LPMO) fold and a His brace with an additional Asp ligand to Cu(II) (see Fig. 12 ▸). X325 proteins seemed initially to be promising candidates for copper-dependent enzymes specialized in the degradation of polysaccharides, and indeed share many structural features with LPMOs. However, they appear to have novel roles related to fungal morphogenesis, pathogenesis and plant symbiosis which are likely to be mediated by their ability to bind copper.

Teze *et al.* (2020 ▸) studied *N*-acetyl­galactosaminidases from the human gut symbiont *Akkermansia muciniphila*, which has been shown to be important for human health. The authors could show that the studied enzymes display dual α-retaining and β-inverting activities, and with structural and bioinformatics analyses explain the dual activity by identifying a flexible loop further explaining the enzyme function. The structure was determined in 2019 at BioMAX to 2.1 Å resolution using molecular replacement.

The development of the BioMAX fragment screening facility FragMAX (Lima *et al.*, 2020 ▸) started in 2018. Its aim is to cover the process from sample preparation to automated data collection, data processing and hit identification. During its commissioning stage, FragMAX ran six user projects, both academic and industrial. One example is the collaborative project between Philipps-Universität Marburg, Helmholtz-Zentrum Berlin, Freie Universität Berlin and MAX IV Laboratory screening the new fragment library, F2X-Entry, against two protein targets (Wollenhaupt *et al.*, 2020 ▸). In this experiment each protein was screened with the entire library of 96 compounds, in the presence and absence of DMSO, using FragMAX standard collection mode (Lima *et al.*, 2020 ▸), in order to verify the hit-rate of the newly developed library with model targets. During eight shifts (8 h each), a total of 572 datasets were collected resulting in 304 structures of hits deposited in the PDB database (PDB group accession codes: G_1002147, G_1002120, G_1002119, G_1002115). This project confirms the high-throughput potential for large data collections at BioMAX. FragMAX opens for users in 2020.

BioMAX has been used for a number of serial crystallography experiments including both the HVE injector and fixed target scanning (Andersson *et al.*, 2019 ▸; Shilova *et al.*, 2020 ▸). While the oscillation data collections in most cases uses a defocused beam and a flux-reducing aperture to set the beam size, the serial crystallography experiments take full advantage of the full flux of the fully focused beam.

Another scientific field that BioMAX has already served is chemical crystallography. Cravcenco *et al.* (2019 ▸) designed a novel multi-chromophoric system, a donor–bridge–acceptor molecule, that was synthesized and structurally characterized at atomic resolution. The donor–bridge–acceptor molecule is able to efficiently mediate excitation energy between states of different multiplicity. This enables higher control of excitation energy transfer pathways and is of importance for advanced functions such as multiplicity conversion in future molecular materials. Data collected at BioMAX allowed much weaker reflections to be recorded, but procedures for data format conversions and interfacing with chemical crystallography data analysis software are required to fully benefit from the data quality.

## 10. Discussion   

In user operation since 2017, BioMAX is the first operational MX beamline at a fourth-generation storage-ring light source. BioMAX is a highly automated beamline with state-of-the-art instrumentation providing optimal performance for most macromolecular crystallography experiments in a wide energy range. The high brilliance X-ray source gives a parallel beam with high flux even when focused down to 20 µm × 5 µm. Most of the functionality of BioMAX is now in operation.

A new microfocus MX beamline called MicroMAX is planned to come into operation in 2022. It is designed in particular for serial crystallography but will also have an optimized experiment setup for oscillation data collection. Serial crystallography makes it easy to collect data from a large number of crystals with important applications being data collection from samples that only form microcrystals, determination of room-temperature structures and time-resolved experiments. We have already started developing serial crystallography at BioMAX with the high-viscosity extruder injector and different fixed target sample holders using the MD3 (Shilova *et al.*, 2020 ▸). BioMAX will also soon be equipped with a Roadrunner (Roedig *et al.*, 2017 ▸) fixed target scanner that will be able to collect data at >100 Hz with sample holders holding >10000 crystals.

BioMAX and MicroMAX with their supporting facilities and infrastructure will provide an excellent facility for a wide range of experiments including high-throughput crystallography, fragment screening, phasing experiments in a wide energy range, serial crystallography with different sample environments and X-ray beams from 1 µm × 1 µm and larger, and time-resolved crystallography.

## Figures and Tables

**Table 1:** MAX IV 3 GeV storage ring and BioMAX main specifications

Storage ring energy	3 GeV
Nominal beam current	500 mA
Electron beam emittance	0.3 nm rad (horizontal) × 8 pm rad (vertical)
Insertion device	In-vacuum undulator (18 mm period)
Monochromator	Si (111), horizontally deflecting double-crystal (26 m from the source)
Focusing optics	KB mirror pair (VFM at 31.5 m and HFM at 32.1 m from the source)
Energy/wavelength range	5–25 keV/0.5–2.5 Å
Focused beam size	20 µm × 5 µm (FWHM, horizontal × vertical)
Flux at sample	2 × 10^13^ photons s^−1^ at 1 Å expected at 500 mA beam current (37 m from the source)
Goniometer	MD3 vertical rotation axis, mini-Kappa
Detector	EIGER 16M photon-counting hybrid pixel detector
Sample changer	ISARA, 29 pucks (Unipuck)

**Table 2:** BioMAX undulator main parameters

Magnet material	NdFeB with *B*_r_ of 1.266 T (specified 1.24 T)
Pole material	Vanadium permendur
Energy range	5–25 keV
Period length	18 mm
Number of periods	111
Minimum magnetic gap	4.2 mm
*K* value at minimum gap	2.19 (specified >1.92)
Tapering of peak field	Up to 5% per meter
Maximum RMS phase error at all gaps	≤ 2.5°

**Table 3:** Mirror specifications for the vertical focusing mirror (VFM) and the horizontally focusing mirror (HFM)

Mirror bulk material	Si
Mirror surface materials	Si, Rh, Pt
Length of mirrors	450 mm (350 mm optical length)
Grazing-incidence angle	3 mrad
VFM shape	Pre-shaped to form an elliptic cylinder at nominal curvature using a one-moment mechanical bender
VFM slope errors	0.3/0.18 µrad (specification/measured)
	0.18/0.14 µrad over 200 mm
VFM roughness	3 Å/2 Å (specification/measured)
HFM shape	Flat, one-moment mechanical bender giving a spherical cylinder
HFM slope errors	0.3/0.29 µrad (specification/measured)
	0.14 µrad measured over 250 mm
HFM roughness	2.5 Å/2 Å (specification/measured)
